# Inhibition of DNA and protein synthesis and cell division by photoactivated haematoporphyrin derivative in hamster ovary cells.

**DOI:** 10.1038/bjc.1986.44

**Published:** 1986-02

**Authors:** G. S. Lin, A. A. Al-Dakan, D. P. Gibson

## Abstract

**Images:**


*Br. J. Cancer* (1986), **53**, 265–269

### Inhibition of DNA and protein synthesis and cell division by photoactivated haematoporphyrin derivative in hamster ovary cells

G.S. Lin*, A.A. Al-Dakan & D.P. Gibson

*Department of Biological and Medical Research, King Faisal Specialist Hospital and Research Centre, Riyadh 11211, Saudi Arabia*

**Summary** Experiments were performed on cultured Chinese hamster ovary cells exposed to haematoporphyrin derivative (HpD) plus light, yielding survival rates of 40–100%. [$^3$H]-thymidine, [$^3$H]-tryptophan and [$^{14}$C]-lysine incorporation were used to quantitate DNA and protein synthesis in surviving cells after exposure. Multiple experiments demonstrated 78% reduction in DNA sysnthesis during the first day after exposure to 20 $\mu$g ml$^{-1}$ HpD plus 1140 J m$^{-2}$ light followed by progressive recovery to the normal rate after 4–6 days. Protein synthesis was somewhat less sensitive dropping by 54% initially and fully recovering by day 4. Although this cell line has a normal cycle time averaging $\sim$15 h, cell division was rarely observed among lone surviving cells until 72 h after exposure. No inhibition was observed in cells exposed to HpD in the dark. These results indicate that photoactivated HpD has a wide spectrum of reversible nuclear and cytoplasmic effects even at sublethal doses. This is consistent with the notion that clinical photodynamic therapy is not likely to result in chronic morbidity.

The photosensitizing drug haematoporphyrin derivative (HpD) and light are demonstrating increasing promise in photodynamic therapy (PDT) for cancer (Dougherty, 1984). Unlike most other antineoplastic agents which act on intracellular metabolic and reproductive pathways taking time to kill target cells, photoactivated HpD can kill cells within minutes probably through production of singlet oxygen which lyses the cell membrane (Bellnier & Dougherty, 1982; Weishaupt *et al.*, 1976).

However, there is growing evidence that HpD plus light may have sublethal effects on other intracellular components in surviving cells. Haematoporphyrin (the less potent parent compound) and HpD plus light have been implicated in induction of sister chromatid exchange and chromosomal aberrations, but not in mutations (Gomer *et al.*, 1983; Evensen & Moan, 1982; Moan *et al.*, 1980). Cells experienced delay of progression through metaphase after treatment with HpD plus light (Christensen, 1981), division delay with depression of DNA synthesis (Moan *et al.*, 1983), and variation in sensitivity through different phases of the cell cycle (Christensen *et al.*, 1981). Photoactivated HpD has been shown to catalyse the breakdown of polynucleotides (Blazek, 1984). At the ultrastructural level it induces profound degenerative changes in mitochondria, ribosomes, endoplasmic reticulum and nuclear chromatin (Kato *et al.*, 1984; Moan *et al.*, 1982), and inhibits mitochondrial cytochrome *c* oxidase activity (Gibson & Hill, 1983). All effects were directly correlated with HpD dose and light exposure level.

In the present work, the effect of sublethal doses of photoactivated HpD on DNA and protein synthesis and on progression through the cell cycle have been studied in cultured Chinese hamster ovary (CHO) cells.

Correspondence: G.S. Lin.
*Present address: Department of Biophysics, All-India Institute of Medical Sciences, New Delhi 110029, India.
Received 16 April 1985; and in revised form, 12 September 1985.

### Materials and methods

CHO cells (CHO–Kl from American Type Culture Collection) were cultured in McCoy's 5A nutrient medium with 10% foetal bovine serum, 100 IU ml$^{-1}$ penicillin G and 100 $\mu$g ml$^{-1}$ streptomycin. They were maintained in 25-cm$^2$ tissue culture flasks with 5 ml medium and kept at 37°C in a humidified 5% $CO_2$ incubator.

HpD was synthesized from haematoporphyrin (Calbiochem) by the method of Lipson *et al.* (1961), sterilized through a 0.22 $\mu$m Millipore filter, and pre-tested for biological activity. Cells were illuminated in a vertical incubator to maintain an ambient temperature of 34–35°C. Cells were exposed through the bottom of the flasks in an inverted illuminator with plexiglass table at a dose rate of $1.90 \pm 0.05$ W m$^{-2}$. The tungsten light was filtered through heat absorbing glass, and the temperature of the medium was monitored with a thermistor probe and controlled to within 36–38°C.

© The Macmillan Press Ltd., 1986

CHO cells were plated 3–4 days in advance of the experiment to provide stable log phase cultures at the time of exposure to HpD and light. Cells to be assayed on day 0 were plated at 5000 cells per tissue culture flask, decreasing to 500 cells/flask for cells to be assayed on day 6 to avoid confluence. They were then incubated with 0–60 $\mu g\, ml^{-1}$ HpD for 90 min. Cells were exposed to 1140 $J\, m^{-2}$ incandescent light over 10 min through the bottom of the tissue culture flasks at 36–38°C on day 0. The cultures were washed once immediately after exposure to HpD and light with Hank's balanced salt solution (HBSS), and then fresh medium was added.

The cells were then assayed on days 0, 1, 2, 3, 4 and 6 for DNA synthesis by addition of 1.0 or 5.0 $\mu Ci\, ml^{-1}$ [$^3$H]-thymidine, and protein synthesis by addition of 2.0 $\mu Ci\, ml^{-1}$ [$^3$H]-tryptophan or 0.2 $\mu Ci\, ml^{-1}$ [$^{14}$C]-lysine (for double labelling with [$^3$H]-thymidine). The cells were incubated at 37°C with the radioactive labels for 16 h, then washed twice with HBSS. After 3 h of incubation in fresh nutrient medium, cell viability among attached cells was determined by dye exclusion using trypan blue and visual counting of cells which were or were not stained. Cells were then washed thrice with HBSS. The remaining attached cells were dissolved in 1.0 M sodium hydroxide (NaOH) for 3 h with periodic vortex mixing. Duplicate aliquots of 0.5 ml each were added to 4.5 ml of Biofluor scintillation emulsifier cocktail (New England Nuclear), vortex mixed, and read in a Packard Tricarb liquid scintillation counter. Cellular protein content was measured by absorbances at 260 nm and 280 nm on a Gilford Spectrophotometer 250 by the method of Warburg and Christian (1942).

### Results

Cells were exposed to 0, 5, 10 and 20 $\mu g\, ml^{-1}$ HpD, 1140 $J\, m^{-2}$ light, HpD alone, and neither. These levels of HpD plus light yielded a survival rate of 40–100%. Relative uptake of thymidine, tryptophan and lysine was quantitated in terms of c.p.m. and mg protein in surviving cells:

$$\text{uptake} = \frac{\text{c.p.m.}}{(\text{mg protein}) \times (\text{surviving fraction})}.$$

Although total cellular protein content may be slightly affected by an altered rate of protein synthesis, differences in uptake can only be slightly underestimated but never exaggerated. DNA synthesis is shown in terms of [$^3$H]-thymidine incorporation relative to untreated control cultures in Figure 1. Multiple experiments demonstrated a

**Figure 1** DNA synthesis relative to untreated control (●) over time following exposure to 5 $\mu g\, ml^{-1}$ HpD plus light (▲), 10 $\mu g\, ml^{-1}$ HpD plus light (▼), and 20 $\mu g\, ml^{-1}$ HpD with light (■) and without light (◆). Bars represent standard errors of 3–8 experiments.

78% reduction in DNA synthesis during the first 16 h (day 0) following exposure to 20 $\mu g\, ml^{-1}$ photoactivated HpD. This inhibition was followed by progressive recovery to the normal rate by day 6. The data suggest a small over-production of DNA during the final phase of recovery on day 4, although this deviation from the normal rate was not statistically significant ($P > 0.2$ by the two-tailed $t$-test). The same general pattern with reduced deviation from normal was seen for smaller doses of HpD, except that the over-correction occurred on day 3 and full normalization was achieved on day 4. Enhanced DNA synthesis was observed during the first 3 days after 20 $\mu g\, ml^{-1}$ HpD in the dark, although this elevation was also not statistically significant ($P > 0.05$).

Protein synthesis is illustrated in terms of incorporation of radiolabelled amino acids relative to untreated control cultures in Figure 2. Protein synthesis was somewhat less sensitive dropping initially by 54% after exposure to 20 $\mu g\, ml^{-1}$ HpD plus light, and fully recovering by day 4. Inhibition of protein synthesis at 5 $\mu g\, ml^{-1}$ HpD was not detectable. Over-correction following inhibition was negligible. Protein synthesis was not affected by HpD alone.

The comparative sensitivity of CHO cells with respect to DNA synthesis, protein synthesis, and cell survival is summarized in Figure 3. DNA synthesis was clearly the most sensitive to inhibition by photoactivated HpD, followed closely by protein

**Figure 2** Protein synthesis relative to untreated control (●) over time following exposure to 5 $\mu g\,ml^{-1}$ HpD plus light (▲), 10 $\mu g\,ml^{-1}$ HpD plus light (▼), and 20 $\mu g\,ml^{-1}$ HpD with light (■) and without light (◆). Bars represent standard errors of 3–8 experiments.

**Figure 3** Comparative sensitivity of log phase CHO cells to inhibition of DNA (■) and protein synthesis (▲) and survival (●) when assayed 3 h after exposure to HpD and light.

synthesis (dose ratio of 1.2 at the 50% inhibition level). The dose ratio between 50% survival and 50% inhibition of DNA synthesis was 1.9.

Cells in small colonies were exposed to 30 $\mu g\,ml^{-1}$ HpD plus 1140 $J\,m^{-2}$ light to achieve survival levels of 1–3%. Figure 4 shows solitary surviving cells in a field of otherwise dead cells. Although this cell line has a normal log phase cycle time averaging ~15 h, cell division was rarely observed among lone surviving cells until at least 72 h after exposure. No division delay was observed in cells exposed to HpD without light.

**Figure 4** Division delay following treatment with 30 $\mu g\,ml^{-1}$ HpD plus light is demonstrated by (a) a single surviving cell in a colony of lysed cells after 72 h (85×), and (b) a surviving cell which has just completed its first mitotic division after 72 h (210×). Phase contrast.

### Discussion

The present results corroborate previous studies on NHIK 3025 human carcinoma cells demonstrating that DNA synthesis is the most sensitive to suppression by photoactivated HpD, followed by protein synthesis, and then cell survival (Moan *et al.*, 1983). Repair of HpD-induced sublethal damage which cumulatively results in lysis of the cell membrane has been studied using a split-dose technique in human bladder carcinoma cells (Bellnier *et al.*, 1984). Recovery from division delay was reliably achieved after several generation times with no irreversible loss of proliferative capacity. The evidence collectively demonstrates that cultured mammalian cells can effectively repair and recover from sublethal damage induced by photoactivated HpD.

Primary inhibition of DNA synthesis by sublethal doses of HpD plus light suggests at least partial blockage or delay of the cell cycle in S phase. This could account for division delay as well as reduced

**Figure 5** Cluster of lymphoblasts demonstrating elevated red HpD fluorescence localization in nucleoli under green light. 320 ×.

protein synthesis associated with slower cycling and growth. Coincident recovery from inhibition of DNA and protein synthesis and division delay 3–4 days after treatment suggests that these effects may be coupled. Alternatively, HpD-induced suppression of enzymatic activity, as was shown for cytochrome *c* oxidase (Gibson & Hill, 1983), may be a generalized phenomenon. If so, photoactivated HpD may simultaneously inhibit different enzymes involved in DNA replication, protein translation, and progression through the cell cycle (particularly S and M phases). However, coincident recovery would not necessarily be anticipated.

Since HpD damage is probably through production of singlet oxygen which diffuses only $\sim 0.1\,\mu m$ in a cell before decaying (Moan *et al.*, 1979), HpD damage is essentially limited to the intracellular sites of HpD localization. According to the pattern of intracellular fluorescence in some cells, cell-bound HpD appears to be concentrated in the nucleus. While this is not obvious in CHO cells where the fluorescence appears more uniformly distributed throughout the cell, we have observed the highest concentration of HpD fluorescence in the nucleolus of rapidly cycling human lymphoblasts derived from a patient with non-Hodgkin's lymphoma (Figure 5). This is somewhat different from the nuclear membrane localization of HpD fluorescence observed by Evensen and Moan (1982) in NHIK 3025 cells. It is possible that HpD binds preferentially to nuclear chromatin which is seen electron microscopically to be peripherally distributed in the NHIK 3025 nucleus (Moan *et al.*, 1982), but more distributed throughout the nucleus of human lymphoblasts observed above. Nuclear localization of HpD – whether at the nuclear membrane, in chromatin, or at the nucleolus – could account for the greatest sensitivity of DNA synthesis to inhibition. Despite this, photoactivated HpD is much less active in DNA damage and mutation than ionizing radiations at comparable survival levels (Evensen & Moan, 1980; Gomer *et al.*, 1983; Moan *et al.*, 1980). This emphasizes a critical distinction between reversible inhibition and cellular damage in assessing the effects of HpD treatment.

These results indicate that photoactivated HpD has a wide spectrum of nuclear and cytoplasmic biological activity even at sublethal doses, but full recovery of surviving cells from cyclostasis is reliably achieved after several days. This is consistent with the notion that sublethal effects during clinical PDT are not likely to result in chronic morbidity.

**References**

BELLNIER, D.A. & DOUGHERTY, T.J. (1982). Membrane lysis in Chinese hamster ovary cells treated with hematoporphyrin derivative plus light. *Photochem. Photobiol.*, **36**, 43.

BELLNIER, D.A., PROUT, G.R. & LIN, C-W. (1984). Recovery from damage induced by hematoporphyrin derivative plus light in cultured human bladder carcinoma cells. In *Porphyrin Photosensitization Workshop*, p. 3. Wayne State University: Detroit.

BLAZEK, E.R. (1984). Chemistry of HpD-photosensitized damage in a DNA model compound. *Photochem. Photobiol.*, **39** suppl, 100S.

CHRISTENSEN, T. (1981). Multiplication of human NHIK 3025 cells exposed to porphyrins in combination with light. *Br. J. Cancer*, **44**, 433.

CHRISTENSEN, T., FEREN, K., MOAN, J. & PETTERSEN, E. (1981). Photodynamic effects of haematoporphyrin derivative on synchronized and asynchronous cells of different origin. *Br. J. Cancer*, **44**, 717.

DOUGHERTY, T.J. (1984). Photoradiation therapy. *Urology*, **23** (suppl 3), 61.

EVENSEN, J.F. & MOAN, J. (1982). Photodynamic action and chromosomal damage: a comparison of haematoporphyrin derivative (HpD) and light with X-irradiation. *Br. J. Cancer*, **45**, 456.

GIBSON, S.L. & HILL, R. (1983). Photosensitization of mitochondrial cytochrome c oxidase by hematoporphyrin derivative and related porphyrins in vitro and in vivo. *Cancer Res.*, **43**, 4191.

GOMER, C.J., RUCKER, N., BANERJEE, A. & BENEDICT, W.F. (1983). Comparison of mutagenicity and induction of sister chromatid exchange in Chinese hamster cells exposed to hematoporphyrin derivative photoradiation, ionizing radiation, or ultraviolet radiation. *Cancer Res.*, **43**, 2622.

KATO, H., KONAKA, C., ONO, J. & 10 others. (1984). Cytomorphological changes of HpD and photodynamic therapy. In *Porphyrin Photosensitization Workshop,* p. 28. Wayne State University: Detroit.

LIPSON, R.L., BALDES, E.J. & OLSEN, A.M. (1961). The use of a derivative of hematoporphyrin in tumor detection. *J. Natl. Cancer Inst.,* **26,** 1.

MOAN, J., JOHANNESSEN, J.V., CHRISTENSEN, T., ESPEVIK, T. & McGHIE, J.B. (1982). Porphyrin-sensitized photoinactivation of human cells in vitro. *Am. J. Path.,* **109,** 184.

MOAN, J., McGHIE, J. & JACOBSEN, P.B. (1983). Photodynamic effects on cells in vitro exposed to hematoporphyrin derivative and light. *Photochem. Photobiol.,* **37,** 599.

MOAN, J., PETTERSEN, E.O. & CHRISTENSEN, T. (1979). The mechanism of photodynamic inactivation of human cells in vitro in the presence of haematoporphyrin. *Br. J. Cancer,* **39,** 398.

MOAN, J., WAKSVIK, H. & CHRISTENSEN, T. (1980). DNA single-strand breaks and sister chromatid exchange induced by treatment with hematoporphyrin and light or by X-rays in human NHIK 3025 cells. *Cancer Res.,* **40,** 2915.

WARBURG, O. & CHRISTIAN, W. (1942). Isolierung und Kristallisation des Garungsferments Enolase. *Biochem. Z.,* **310,** 384.

WEISHAUPT, K.R., GOMER, C.J. & DOUGHERTY, T.J. (1976). Identification of singlet oxygen as the cytotoxic agent in photoinactivation of a murine tumor. *Cancer Res.,* **36,** 2326.


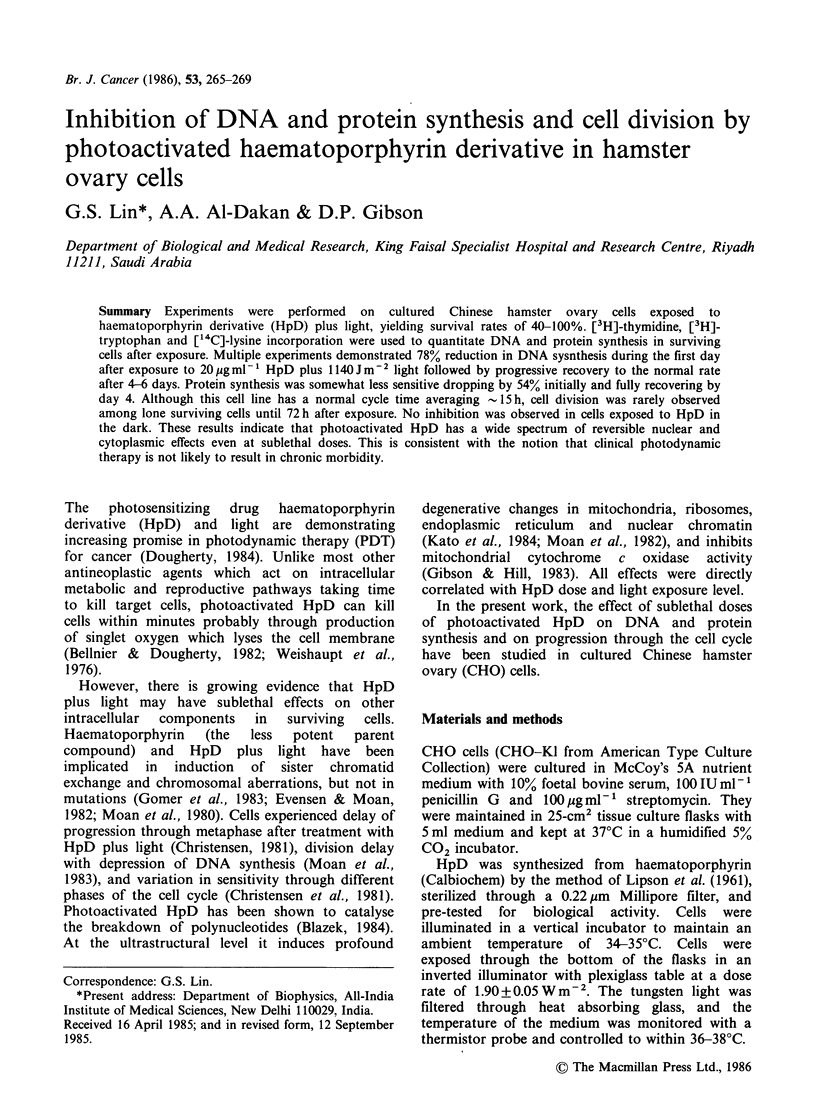

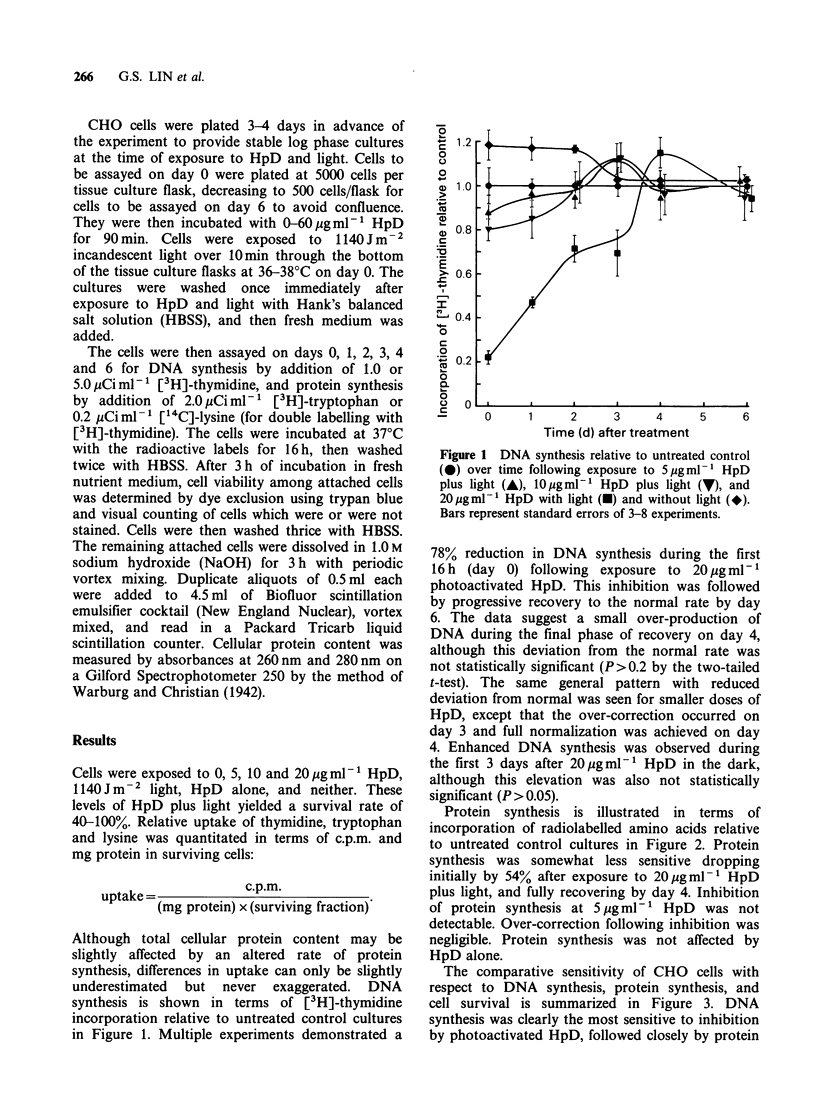

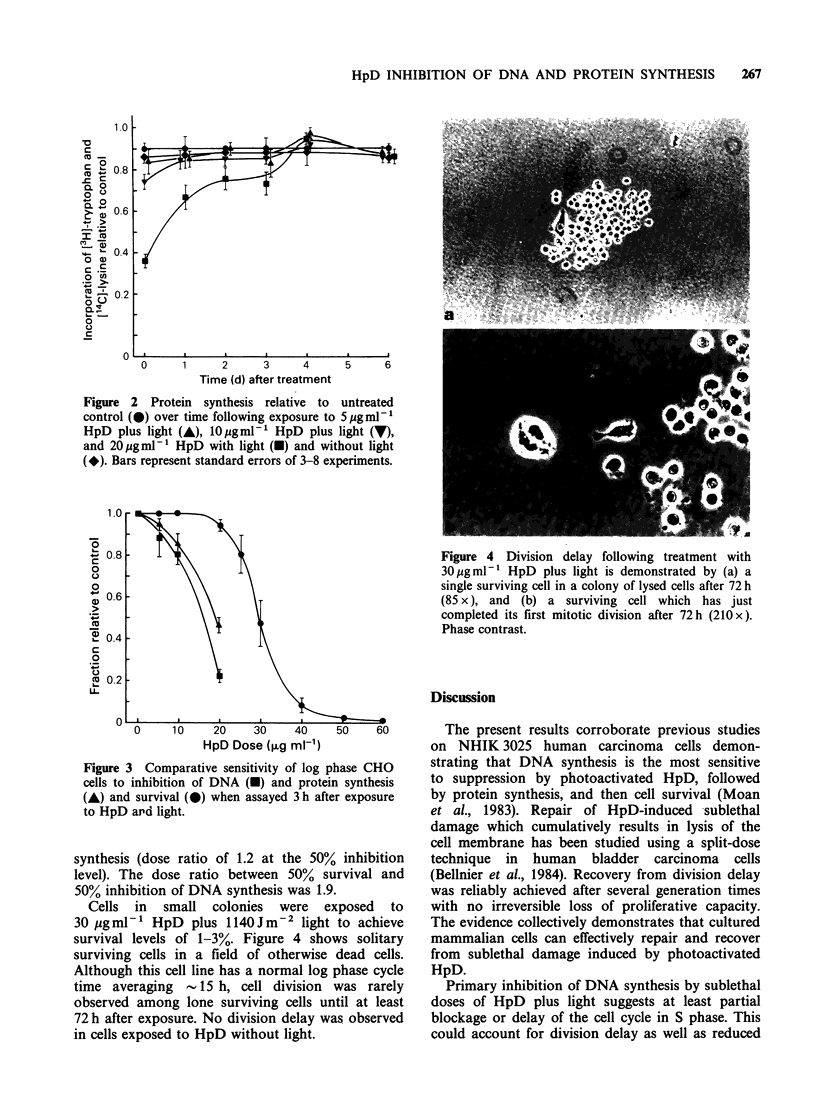

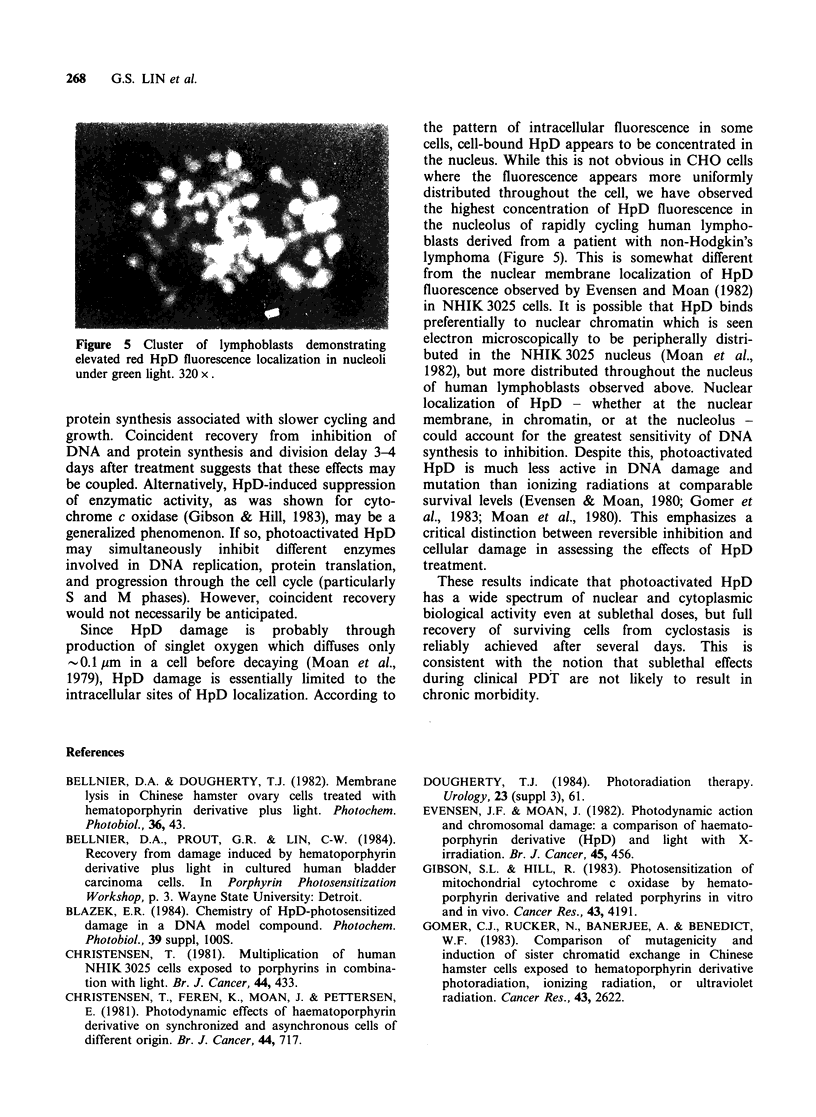

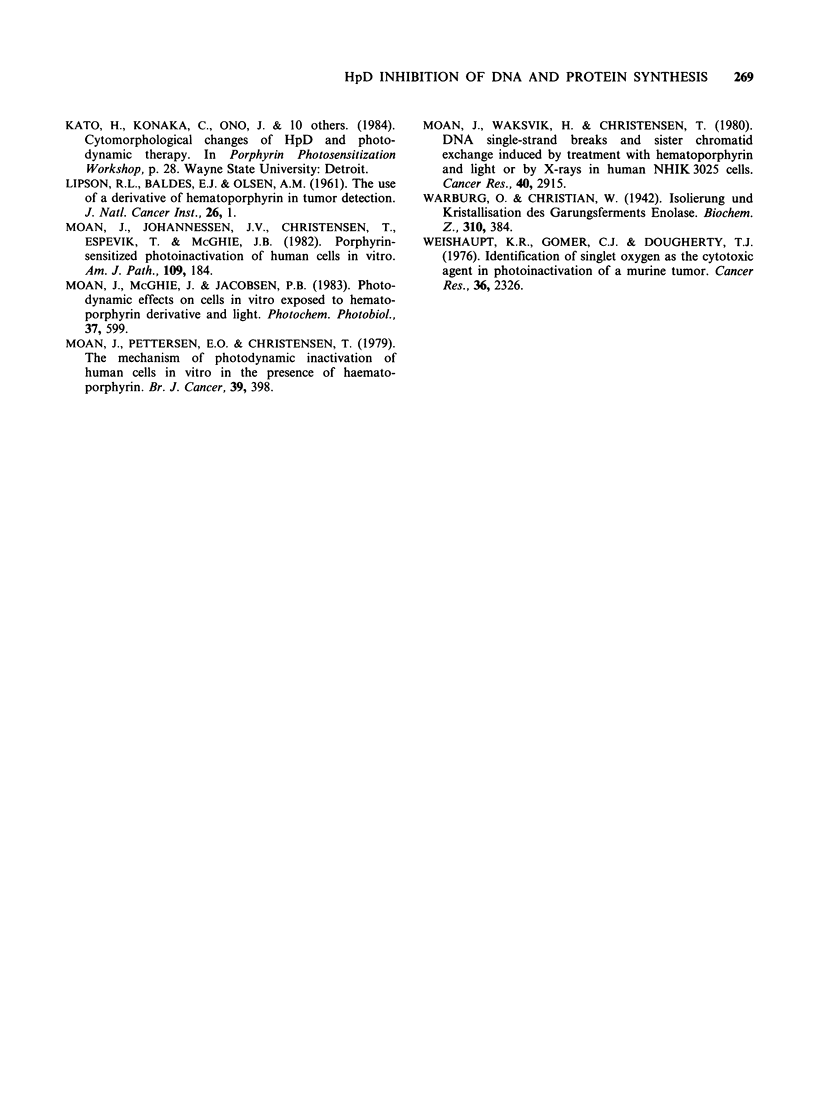


## References

[OCR_00425] Bellnier D. A., Dougherty T. J. (1982). Membrane lysis in Chinese hamster ovary cells treated with hemtoporphyrin derivative plus light.. Photochem Photobiol.

[OCR_00448] Christensen T., Feren K., Moan J., Pettersen E. (1981). Photodynamic effects of haematoporphyrin derivative on synchronized and asynchronous cells of different origin.. Br J Cancer.

[OCR_00443] Christensen T. (1981). Multiplication of human NHIK 3025 cells exposed to porphyrins in combination with light.. Br J Cancer.

[OCR_00454] Dougherty T. J. (1984). Photoradiation therapy.. Urology.

[OCR_00458] Evensen J. F., Moan J. (1982). Photodynamic action and chromosomal damage: a comparison of haematoporphyrin derivative (HpD) and light with X-irradiation.. Br J Cancer.

[OCR_00464] Gibson S. L., Hilf R. (1983). Photosensitization of mitochondrial cytochrome c oxidase by hematoporphyrin derivative and related porphyrins in vitro and in vivo.. Cancer Res.

[OCR_00470] Gomer C. J., Rucker N., Banerjee A., Benedict W. F. (1983). Comparison of mutagenicity and induction of sister chromatid exchange in Chinese hamster cells exposed to hematoporphyrin derivative photoradiation, ionizing radiation, or ultraviolet radiation.. Cancer Res.

[OCR_00486] LIPSON R. L., BALDES E. J., OLSEN A. M. (1961). The use of a derivative of hematoporhyrin in tumor detection.. J Natl Cancer Inst.

[OCR_00491] Moan J., Johannessen J. V., Christensen T., Espevik T., McGhie J. B. (1982). Porphyrin-sensitized photoinactivation of human cells in vitro.. Am J Pathol.

[OCR_00497] Moan J., McGhie J., Jacobsen P. B. (1983). Photodynamic effects on cells in vitro exposed to hematoporphyrin derivative and light.. Photochem Photobiol.

[OCR_00503] Moan J., Pettersen E. O., Christensen T. (1979). The mechanism of photodynamic inactivation of human cells in vitro in the presence of haematoporphyrin.. Br J Cancer.

[OCR_00509] Moan J., Waksvik H., Christensen T. (1980). DNA single-strand breaks and sister chromatid exchanges induced by treatment with hematoporphyrin and light or by x-rays in human NHIK 3025 cells.. Cancer Res.

[OCR_00521] Weishaupt K. R., Gomer C. J., Dougherty T. J. (1976). Identification of singlet oxygen as the cytotoxic agent in photoinactivation of a murine tumor.. Cancer Res.

